# Evaluation of GWAS candidate susceptibility loci for uterine leiomyoma in the multi-ethnic NIEHS uterine fibroid study

**DOI:** 10.3389/fgene.2015.00241

**Published:** 2015-07-14

**Authors:** Brahim Aissani, Kui Zhang, Howard Wiener

**Affiliations:** ^1^Department of Epidemiology, University of Alabama at Birmingham School of Public HealthBirmingham, AL, USA; ^2^Department of Biostatistics, University of Alabama at Birmingham School of Public HealthBirmingham, AL, USA

**Keywords:** uterine fibroids, tumor size, thrombospondin, extracellular matrix, meta-analysis, genetic association, African American, reproductive medicine

## Abstract

We evaluated the association of 56 candidate SNPs identified in two published genome-wide association studies (GWAS) of uterine leiomyoma (UL), or fibroids, with the risk and tumor size in the multi-ethnic uterine fibroid study (NIEHS-UFS). The selected SNPs were genotyped in 916 premenopausal women of African American (AA) and European American (EA) descents and their association with the outcomes was evaluated in race-stratified models and in meta-analysis of risk in NIEHS-UFS and discovery and replication GWAS in the Japanese population. We report moderate associations of variant rs4954368 in *THSD7B* (thrombospondin, type I, domain containing 7B) with tumor size in pooled analysis of AA and EA samples (*P* = 0.004), and at *TNRC6B* (trinucleotide repeat containing 6B) variants rs138039 and rs139909 in EA (*P* = 0.001 and 0.008, respectively). The most significant associations with risk in meta-analysis were observed at *TNRC6B* variants rs739182 (*P* = 3.7 × 10^−10^) and rs2072858 (*P* = 1.1 × 10^−9^) and were stronger than those reported in the discovery GWAS (*P* = 2.01 × 10^−8^ and 2.58 × 10^−8^, respectively). The present study failed to replicate the associations reported for *CCDC57* and *FASN* in a discovery GWAS in populations of European descent. Consistent with previous replication studies in the Right From the Start Study (RFTS) and the BioVU DNA repository, we provide independent evidence for association of *TNRC6B* with both risk and size of UL. The present study is the first to report a replicated association of *THSD7B* with UL, albeit with tumor size and not with risk.

## Introduction

Uterine leiomyoma (UL), or fibroids, are common hormone-dependent tumors that arise in the smooth muscle cells of the uterus. These tumors develop in >75% of North American women over the reproductive age and become clinically apparent in about one-third (Cramer and Patel, 1990). Although benign, UL cause significant gynecologic morbidities including menorrhagia, pelvic pain, urinary incontinence, infertility, and pregnancy complications (Stewart, 2001; Walker and Stewart, 2005). Uterine fibroids are the primary indication for hysterectomy, with an incidence rate of 5.5 per 1000 women in the United States and accounting for about $9.4 billion of public health burden (Farquhar and Steiner, 2002).

Estrogen and progesterone are believed to promote the growth of these tumors and long-term unopposed estrogens are associated with risk (Andersen, 1996). Factors influencing gonadal steroid metabolisms, such as obesity, diet, or exercise show apparent association with risk (Schwartz et al., 2000). Age (increasing risk with increasing premenopausal age), menopause (risk decreases with menopause), and African American (AA) ethnicity (higher risk compared with that of non-Hispanic Whites), which was originally tested in NIEHS-UFS (National Institute of Environmental Health Sciences-Uterine Fibroid Study) (Baird et al., 2003) are established risk factors. The basis for the higher prevalence among African Americans is not well-understood. The genetic contribution to this ethnic disparity was investigated in admixture-based genome-wide scans of the AA population in BWHS (Black Women's Health Study) (Wise et al., 2012) and recently in NIEHS-UFS (Zhang et al., 2015). Both the BWHS (Wise et al., 2012) and NIEHS-UFS found a decreased mean percentage of European ancestry in UL cases compared to controls. The negative correlation between European ancestry and UL prevalence suggests that genetic factors may explain the different prevalence between AA and European American (EA) women.

Genetic predisposition to UL was evidenced in early familial aggregation studies (Vikhlyaeva et al., 1995; Sato et al., 2002). Nonetheless, no convergent views on a potential mechanism of pathogenesis have emerged from the genome linkage scans and genome-wide association studies (GWAS) reported to date (Gross et al., 2004; Cha et al., 2011; Eggert et al., 2012; Wise et al., 2012). These GWA studies pointed to *TNRC6B* (trinucleotide repeat containing 6B) and *BET1L* (Bet1 golgi vesicular membrane trafficking protein-like) as the most significant candidate genes in the Japanese population (Cha et al., 2011) and to *FASN* (fatty acid synthase) and the nearby *CCDC57* (coiled-coil domain containing 57) gene in the European American population of the WGHS (Women Genome Health study) (Eggert et al., 2012) and Australian populations. Furthermore, genetic association studies of UL in European American women enrolled in the Right From The Start (RFTS) cohort and the Biorepository at Vanderbilt University (BioVU) study reported replications of the associations with *BET1L* and *TNRC6B* (Edwards et al., 2013a,b). Recently, in an independent follow-up study to published linkage (Eggert et al., 2012) and admixture (Wise et al., 2012) signals for UL, we reported association data in NIEHS-UFS implicating variants in genes encoding components of the extracellular matrix (ECM) (Aissani et al., 2014), consistent with the current paradigm for altered ECM in UL pathogenesis.

Whole genome sequencing of tumors have identified several genes involved in UL pathology (Makinen et al., 2011; Mehine et al., 2013) and cytogenetic analyses detected recurrent chromosomal aberrations like those affecting the high mobility group AT-hook 2 (*HMGA2*) gene on chromosome 12 in band q14.3 in tumorigenesis (Heim et al., 1988; Turc-Carel et al., 1988). In contrast to these somatic mutations, germline mutations affecting highly conserved amino acids have been reported for the fumarate hydratase-encoding gene (*FH*) on chromosome 1q43 in tumor syndromes (Alam et al., 2001; Tomlinson et al., 2002) but only in rare cases of non-syndromic UL (Barker et al., 2002; Kiuru et al., 2002).

Replicating GWAS candidate SNPs in independent studies of populations with different ancestry, especially in the high-risk African American population, is an important undertaking toward validation of the association results reported in discovery GWAS. Another motivation for conducting the present study is the lack of shared candidate SNPs between the two available GWAS. In the present study, we sampled 916 clinically ascertained premenopausal women participants in the multi-ethnic NIEHS-UFS to evaluate the association of candidate SNPs (single nucleotide polymorphisms) from GWA studies conducted in the Japanese (JPN), EA, and Australian (AUS) populations, as well as a few SNPs in or nearby candidate genes, with the risk of UL and tumor size. We report double-sided *p*-values uncorrected for multiple testing from meta-analyses and from race-stratified analyses for the two predominant AA (393 cases and 132 controls) and EA (195 cases and 196 controls) ethnic groups from logistic regression models with or without adjustment for covariates.

## Materials and methods

### Study population

The characteristics of the study population were reported in previous studies (Aissani et al., 2013, 2014). Briefly, women aged 35–51 years, were randomly selected from a computerized list of members of a prepaid urban health plan for enrollment in the NIEHS-UFS (Baird et al., 2003). Self-administered questionnaires were used to collect demographic data. Reproductive and gynecologic history data were collected during a telephone interview. About 92% of the study population was self-identified as AA or non-Hispanic EA. Among the premenopausal women enrolled in the study and who had ultrasound examinations (*n* = 1119), 1045 (93%) had available DNA specimens and were of African American (*n* = 574), non-Hispanic European American (394) or other (*n* = 77) ethnicity. The NIEHS-UFS and the present sub-study were approved by the Human Subject's Review boards at the NIEHS, George Washington University and University of Alabama at Birmingham, respectively. Participants to NIEHS-UFS were fully informed and were enrolled only after full written consents were obtained.

For meta-analysis, model estimates were available only from the GWAS in the Japanese population (Cha et al., 2011). This GWAS sampled 1607 individuals clinically diagnosed with UL and 1328 individual controls for the discovery study, and 3466 clinically diagnosed UL cases 3245 individual controls for the replication study. The GWAS obtained written patient consents in accordance with the Ethical Committee at each of The Institute of Medical Science of the University of Tokyo and the Center for Genomic Medicine of RIKEN.

### Covariates

Covariates included age, age at menarche, parity (pregnancies after age 25; earlier births were not significantly related to fibroid development in the NIEHS-UFS cohort (Dunson and Baird, 2002; Baird and Dunson, 2003), body mass index (BMI), and physical activity.

### Ascertainment

Fibroid status was assessed by ultrasonography at baseline or by medical record review in 80 and 90% of AA and EA participants, respectively. For the study participants who had recent pelvic ultrasound examinations, the radiology records were examined for the presence of fibroids. The remaining participants were asked to have a pelvic ultrasound examination at a primary care site. If neither ultrasounds nor medical record reviews could be conducted, the concerned participants were excluded from the present study. Clinical examinations consisted of both transabdominal and transvaginal ultrasonographies. The abdominal portion evaluated fibroid changes arising from the upper uterus that could be missed by the transvaginal approach alone. Tumor size was classified in three categories (Small, Medium, and Large) measured by the diameter of the tumors (S ≤ 2 cm, 2 cm < M < 4 cm, L ≥ 4 cm). For participants diagnosed with multiple tumors, the largest tumor determined the size category.

No clinical information on the size of fibroids was available in the GWAS sample used in meta-analysis. Compared with the reported family history among cases (21.03 and 16.56% for the discovery and replication samples, respectively), only 5.11 and 1.94% of the controls in the discovery and replication sets, respectively, reported family history of UL (Cha et al., 2011).

### SNP selection and typing

Illumina iSelect assay (Illumina Inc., San Diego, CA) was used to type a total of 63 SNPs selected among the most significantly associated SNPs in GWAS. We also included additional SNPs in associated genes or genes adjacent to them, as well as a small number of SNPs in the candidate *HMGA2*. Reliability in typing data was assessed by inclusion of blind intra- and inter-plate duplicates representative of each of the study population groups. For accurate assessment of African descent, we included a set of 25 unrelated Yoruban (YRI) samples. A total of 2682 SNPs (from an extended set of 4363 SNPs typed in a previous study and also that included 147 ancestry informative markers) that were common to our data and to the majority of the HapMap III reference populations was used to assess ethnic membership of quality control samples. About 35% of the SNPs were from chromosome 1q43; the remaining SNPs have genome-wide distribution (Aissani et al., 2014). All SNPs in the extended set were selected to have a minor allele frequency (MAF) of at least 5% and most of them are inter-population tagging SNPs relevant to the ethnic backgrounds of the NIEHS-UFS study populations (Aissani et al., 2013).

Principal component analysis was used to assess the Japanese ethnicity of the case and control individuals sampled for the GWAS (Cha et al., 2011). The control individuals studied in the discovery and replication GWAS were older than the cases (mean ages 42.5 vs. 48.9 years and 45.2 vs. 62.3 years in discovery and replication sets, respectively). This study design is important to keep downward the rate of misclassification, which can be substantial in case and control studies where controls are not clinically ascertained. The participants to NIEHS-UFS were all ≥35 years of age and about two-thirds were ≥40 years of age.

### Statistical analysis

#### Quality control

SNP calls were checked for adherence to Hardy–Weinberg equilibrium (HWE) in each of the affection status and population stratum, and SNPs deviating (*p* < 0.01) from HWE in UL-free controls were excluded.

#### Analytical design

Association of SNPs with risk of UL and tumor size was evaluated separately in each population stratum using dichotomous and polytomous logistic regression modeling, respectively, in SAS (SAS, Cary, NC), and in meta-analyses by fitting random effect models to the data in the metafor R package (http://www.jstatsoft.org/v36/i03/) (Viechtbauer, 2010). Heterogeneity between studies was assessed by the Cochran's *Q*-test that is part of DerSimonian-Laird estimator in the pooling method (DerSimonian and Laird, 1986). Because the discovery GWAS data were derived from models with no adjustments for the covariates, for comparisons, we report data from both adjusted and unadjusted models in NIEHS-UFS and from unadjusted models in the meta-analysis of NIEHS-UFS and Japanese GWAS.

Previous studies in NIEHS-UFS have assessed the levels of the ordinal covariates that influenced or modified the risk of UL (Baird et al., 1998, 2007; Baird and Dunson, 2003); we therefore modeled these covariates accordingly. Specifically, we modeled age as a continuous variable and BMI as an ordinal variable (based on categories <25, 25–29.99, 30–34.99, 35+). The other covariates were age of menarche modeled as a dichotomous variable (age ≤11 years vs. other ages), exercise as a 4-level ordinal variable (lower third, middle third, next sixth, top sixth as described (Baird et al., 2007) and parity defined as having or not having given birth at age 25 and older (binary variable).

The association of SNP genotypes with UL outcomes was tested in principal component analysis (PCA)-defined groups assuming additive genetic effects as described (Aissani et al., 2013). For the polytomous models, the assumption for proportional odds in both or in either AA or EA study population was checked and reported if not met. UL was modeled as a dichotomous outcome (case-control design comparing UL-free category to the combination of tumor size categories S, M, and L) or as a polytomous outcome comparing UL size categories (S vs. M and L and S and M vs. L).

SNP tests that passed Bonferroni-corrected significance thresholds, which were *p* = 0.05/56 = 9 × 10^−4^ for multiple testing of 56 quality control-filtered SNPs in NIEHS-UFS and genome-wide significance *p* = 10^−7^ in meta-analysis of NIEHS-UFS and discovery GWAS, were deemed to be statistically significant.

## Results

Characteristics of the participants available for testing the genetic association with the risk of UL (case-control design) and tumor size (case-only design) were reported in a previous study (Aissani et al., 2013, 2014). Except for a unique individual of European descent, typing was achieved in the entire study population (*n* = 1045). The call rate was >98% in about 92% of sampled individuals. Among the 1045 typed samples, 42 (4.0%) with call rates <90% were excluded from the analysis; this yielded a total of 1003 samples for analysis. The overall concordance rate between duplicates was 99.6%, implying that a false discovery due to typing errors is unlikely. Discriminant analysis corrected ethnic group inference for several individuals who self-identified as “other populations” to either EA or AA ancestry (Supplementary Figure 1). Sixteen individuals who self-identified as African American (AA) and one individual as a non-Hispanic European American (EA) clustered more closely with the Yoruban African population (YRI). This observation prompted the exclusion of this subset of 17 individuals, resulting in a total of 986 individuals (525 AA, 391 EA, and 70 other) available for analysis.

Of the 63 assayed SNPs, 56 passed the QC filters including adherence to HWE in the control groups. Tables 1, 2 display the results of association with the risk and tumor size outcomes, respectively, for the most significant (Bonferroni-uncorrected *P* ≤ 0.05) SNPs in any of the tested models. Race-stratified analyses and meta-analyses were restricted to the two largest ethnic groups AA and EA. To allow comparisons with the discovery GWAS, which were not adjusted for the covariates modeled in the present study, we report results from models with or without adjustment for covariates (Supplementary Tables S1, S2). The results showed no significant differences between the adjusted and unadjusted models.

**Table 1:** **Association of candidate SNPs ^a^with risk of uterine fibroids in the NIEHS uterine fibroid study**.

**SNP**	**Allele^b^**		**A1 frequency**		**Chr^d^**	**Position, bp**	**Gene/gene region**	**Logistic regression^e^**						**Meta-analysis**	
	**A1**	**A2**	**NIEHS-UFS^c^**					**EA (*****n*** **= 391)**			**AA (*****n*** **= 525)**			**EA + AA (*****n*** **= 916)**	
			**(EA)**	**(AA)**				**Adjusted**		**Un-adjusted**	**Adjusted^f^**		**Un-adjusted**	***P***	***P*-het^g^**
								**OR^f^**	***P***	***P***	**OR**	***P***	***P***		
rs1481046	G	T	0.397	0.360	8	71,371,902	*LOC*^h^	0.90	4.9E-01	7.7E-01	0.65	4.9E-03	2.2E-02	1.5E-01	1.2E-01
rs11601352	A	G	0.042	0.016	11	249,105	*PSMD13*^i^	0.62	2.3E-01	3.9E-01	0.35	4.2E-02	4.2E-02	4.2E-02	9.9E-01
rs2270451	C	T	0.124	0.035	12	12,849,826	*GPR19*^j^	1.50	1.0E-01	8.8E-02	3.51	2.4E-02	4.6E-02	2.2E-02	5.4E-01
rs2301523	A	G	0.130	0.197	22	27,062,669	*MIAT*	0.90	6.4E-01	8.6E-01	1.49	3.9E-02	5.9E-02	6.3E-01	6.4E-02
rs6001794	T	G	0.212	0.219	22	40,529,415	*TNRC6B*	0.66	3.4E-02	3.3E-02	1.05	7.8E-01	8.4E-01	1.8E-01	4.3E-01
rs11089974	T	C	0.216	0.207	22	40,543,608	*TNRC6B*	0.67	4.4E-02	5.9E-02	1.07	7.2E-01	9.4E-01	3.5E-01	4.0E-01
rs739182	T	G	0.338	0.122	22	40,615,276	*TNRC6B*	0.76	8.8E-02	1.6E-01	0.76	2.4E-01	2.7E-01	3.0E-02	5.7E-01
rs2072858	C	T	0.345	0.159	22	40,708,679	*TNRC6B*	1.32	8.4E-02	1.3E-01	1.14	4.9E-01	5.8E-01	2.5E-02	9.7E-01

a *Candidate single nucleotide polymorphisms (SNPs) that passed the threshold of significance in a genome-wide association study (GWAS) of uterine leiomyoma in the Japanese population (Cha et al., 2011)*.

b *A1, Alternate allele; A2, reference allele*.

c *National Institute of Environmental Health Science uterine fibroid study (NIEHS-UFS) in African American (AA) and European American (EA) populations*.

d *Chromosome*.

e *Logistic regression analysis assuming an additive genetic model*.

f *Odds ratios (OR) from models adjusted for age, age at menarche, parity after age 25, body mass index, and physical activity*.

g *p-Value for heterogeneity (P-het) across samples*.

h *About 15 Kb upstream of long-non-coding RNA LOC101926892*.

i *Intronic SNP in PSMD13 (proteasome 26S subunit, non-ATPase, 13) located about 40 Kb from candidate BET1L (Bet1 golgi vesicular membrane trafficking protein-like)*.

j *SNP in the 2-Kb upstream sequence of GPR19 (G protein-coupled receptor 19)*.

*MIAT (myocardial infarction associated transcript); TNRC6B (trinucleotide repeat containing 6B)*.

**Table 2:** **Association of candidate SNPs ^a^ with the size of uterine fibroids in NIEHS uterine fibroid study**.

**SNP**	**Allele^b^**		**A1 frequency**		**Chr^d^**	**Position, bp**	**Gene/gene region**	**Logistic regression^e^**				**Meta-analysis**	
	**A1**	**A2**	**NIEHS-UFS^c^**					**EA (*****n*** **= 195)**		**AA (*****n*** **= 393)**		**EA + AA (*****n*** **= 588)**	
			**(EA)**	**(AA)**				**OR^f^**	***P***	**OR**	***P***	***P***	***P*-het^g^**
rs4503307	C	T	0.041	0.237	1	80,641,525	intergenic	1.15	7.8E-01	1.50	2.1E-02	1.3E-01	2.4E-01
rs4954368	C	T	0.303	0.383	2	137,757,933	*THSD7B*	1.51	4.6E-02	1.34	3.5E-02	4.3E-03	6.6E-01
rs12423095	C	T	0.018	0.047	12	66,291,232	*HMGA2*	0.89	8.6E-01	1.96	3.9E-02	4.7E-01	9.9E-01
rs2226994	G	A	0.351	0.489	16	13,664,942	*LOC*^h^	1.67	1.5E-02	1.00	9.8E-01	4.0E-01	4.3E-02
rs138039	A	G	0.369	0.109	22	40,621,288	*TNRC6B*	1.96	1.3E-03	0.88	5.8E-01	2.1E-01	1.6E-01
rs139909	C	T	0.313	0.084	22	40,697,581	*TNRC6B*	1.73	7.9E-03	0.96	8.7E-01	8.5E-02	2.6E-01

a *Candidate single nucleotide polymorphisms (SNPs) that passed the threshold of significance in a genome-wide association study (GWAS) of uterine leiomyoma (UL) in the Japanese population (Cha et al., 2011), with exception to rs12423095, which was selected from UL candidate gene HMGA2 (high mobility group AT-hook 2)*.

b *A1, Alternate allele; A2, reference allele*.

c *National Institute of Environmental Health Science uterine fibroid study (NIEHS-UFS) in African American (AA) and European American (EA) populations*.

d *Chromosome*.

e *Proportional odds models from logistic regression analysis*.

f *Odds ratios (OR) from logistic regression models adjusted for age, age at menarche, parity after age 25, body mass index, and physical activity*.

g *P-het: P-value for heterogeneity across samples*.

h *LOC101927287; THSD7B (thrombospondin, type I, domain containing 7B) TNRC6B (trinucleotide repeat containing 6B)*.

Race-stratified analyses of risk showed moderate associations (*P* < 0.05) with 6 SNPs, one in each of chromosomes 8, 11, and 12, and 3 on chromosome 22 (Table 1). The most significant association (*P* = 0.005) occurred in the AA group at rs1481046 located about 15 Kb upstream of a long-non-coding RNA (*LOC101926892*) of uncharacterized function. Among the associated SNPs, three locate to or near previously replicated *BETL1* and *TNRC6B* genes (Edwards et al., 2013b). The intronic SNP rs11601352 in *PSMD13* (proteasome 26S subunit, non-ATPase, 13) is located about 40 Kb from the replicated candidate *BET1L*. The moderate association with this SNP was observed only in the AA group and in meta-analysis (*P* = 0.042), whereas the association with the two other SNPs, rs6001794, and rs11089974, in *TNRC6B* was observed in the EA group but not in the meta-analysis. The two other associated SNPs in the AA group, rs2270451 (*P* = 0.024) and rs2301523 (*P* = 0.039), are located in the 2-Kb upstream sequence of *GPR19* (G protein-coupled receptor 19) and within the non-protein coding RNA *MIAT* (myocardial infarction associated transcript), respectively. Two *TNRC6B* intronic SNPs, rs739182, and rs2072858, showed moderate associations (*P* = 0.030 and *P* = 0.025, respectively) but only in meta-analyses; these SNPs are in strong linkage disequilibrium (LD) in EA (*r*^2^ = 0.95; Supplementary Table S4) and in moderate LD in AA (*r*^2^ = 0.58; Supplementary Table S5).

Association with tumor size was tested in case-only designs using proportional odds models and assuming an additive genetic model with adjustments for covariates. No variants that associated with risk also associate with tumor size; however, SNPs in *TNRC6B* other than those affecting risk influenced significantly (*P* = 0.001 at rs138039 and *P* = 0.008 at rs139909) tumor size in EA (Table 2). Examination of the LD pattern in TNRC6B showed that these two SNPs are in LD with each other both in EA (*r*^2^ = 0.74) and AA (*r*^2^ = 0.76) but not with any other tested *TNRC6B* SNP (*r*^2^ < 0.05) in AA (Supplementary Table S5) or in EA (*r*^2^ < 0.35) (Supplementary Table S4).

Variant rs12423095 in *HMGA2* showed a moderate association with tumor size in AA only although no significant heterogeneity was observed between AA and EA (Supplementary Table S2), possibly indicating a spurious association due to the low minor allele frequency at this SNP. Consistent with these data, rs12423095 showed weak or no LD (*r*^2^ < 0.15) with the other tested *HMGA2* SNPs in both study groups. The sole association with tumor size observed separately in AA (*P* = 0.035) and EA (*P* = 0.046), and in meta-analysis (*P* = 0.004) was with the intronic variant rs4954368 in *THSD7B* (thrombospondin, type I, domain containing 7B gene) (Table 2 and Table S2). Of note, given that none of the significantly associated SNPs failed to meet the assumption for proportional odds, these results imply single OR for the contrasted ordinal outcomes.

We re-evaluated the association of the full set of 56 SNPs with risk in meta-analyses of NIEHS-UFS (*n* = 916) and the discovery (*n* = 3035) and replication (*n* = 6711) GWAS in the Japanese population (Cha et al., 2011). The results of the meta-analyses showed significant associations (*P* < 1.3 × 10^−7^) with 5 of the 8 tested SNPs in *TNRC6B* and a moderate association at the candidate *PSMD13*/*BET1L* region (rs11601352, *P* = 0.0047) (Table 3). In particular, *TNRC6B* variants rs739182 (*P* = 3.7 × 10^−10^) and rs2072858 (*P* = 1.1 × 10^−9^) showed stronger associations than in the discovery GWAS (*P* = 2.01 × 10^−8^ and 2.58 × 10^−8^, respectively). No association was observed at the *TNRC6B* variants rs138039 and rs139909 that associated moderately with tumor size in the EA group (Table 2). Examination of the minor allele frequency (MAF) at these two SNPs showed higher MAFs in EA compared to AA, and opposite minor and major alleles in the Japanese population (Table S3).

**Table 3:** **Meta-analysis of risk of uterine leiomyoma among North American and Japanese women**.

**SNP^a^**	**Chr^b^**	**Position, bp**	**allele^c^**		**A1 allele frequency^d^**			**Gene/gene region**	**Meta-analysis**			

			**A1**	**A2**	**EA**	**AA**	**JPN**		**Beta**	**SE^e^**	***P***	***P*-het^f^**
					**(*n* = 525)**	**(*n* = 391)**	**(*n* = 9746)**					
rs991964	2	59,282,591	A	G	0.458	0.165	0.329	*LINC01122*	0.162	0.066	1.5 × 10^−2^	0.044
rs11601352	11	249,105	A	G	0.042	0.016	0.055	*PSMD13*	0.384	0.136	4.7 × 10^−3^	0.067
rs2172873	12	103,125,690	T	C	0.060	0.322	0.203	PAH/I*GF1*	0.165	0.065	1.2 × 10^−2^	0.100
rs2226994	16	13,664,942	A	G	0.367	0.491	0.772	*LOC101927287*	0.125	0.063	4.7 × 10^−2^	0.062
rs6502051	17	80,059,332	A	C	0.481	0.383		*CCDC57*	0.228	0.104	2.9 × 10^−2^	0.300
rs6001794	22	40,529,415	T	G	0.212	0.219	0.358	*TNRC6B*	−0.206	0.039	1.3 × 10^−7^	0.290
rs11089974	22	40,543,608	T	C	0.216	0.207	0.365	*TNRC6B*	−0.185	0.032	7.5 × 10^−9^	0.360
rs739182	22	40,615,276	T	G	0.338	0.122	0.488	*TNRC6B*	−0.166	0.027	3.7 × 10^−10^	0.530
rs12484776	22	40,652,873	G	A	0.208	0.089	0.381	*TNRC6B*	−0.204	0.030	5.7 × 10^−12^	0.590
rs2072858	22	40,708,679	C	T	0.345	0.159	0.489	*TNRC6B*	−0.161	0.026	1.1 × 10^−9^	0.630

a *Candidate single nucleotide polymorphisms (SNPs) that passed the threshold of significance in a genome-wide association study (GWAS) of uterine leiomyoma in the Japanese population (Cha et al., 2011), with exception to the SNP in CCDC57 (coiled-coil domain containing 57 gene) which was selected along with other SNPs (Tables S1 and S2) from a GWAS in European American and Australian populations of the Women's Genome Health Study (WGHS) (Eggert et al., 2012). Note that because estimates of beta and SE from WGHS were not available for the present study, only estimates obtained for the NIEHS-UFS African American (AA) and European American (EA) samples were used in meta-analysis*.

b *Chromosome*.

c *A1, Alternate allele; A2, reference allele*.

d *Alternate allele (A1) frequency in AA, EA, and Japanese (JPN)*.

e *Beta coefficients (beta) and standard errors (SE) from meta-analyses using random-effect models. For consistency with the model used in the GWAS in JPN, beta and SE estimates for the NIEHS-UFS samples were from unadjusted logistic regression models (Table 1 and Table S1)*.

f *P-value for heterogeneity (P–het). LINC01122 (long intergenic non-protein coding RNA 1122); PSMD13 (proteasome 26S subunit, non-ATPase, 13) located about 40 Kb from candidate BET1L (Bet1 golgi vesicular membrane trafficking protein-like); PAH (phenylalanine hydroxylase); IGF1 (Insuline-like growth factor 1); TNRC6B (trinucleotide repeat containing 6B)*.

Moderate heterogeneities between the studies were observed at three gene loci including *PSMD13*/*BET1L* but not at the significantly associated *TNRC6B* or at the moderately associated *CCDC57*. Of note, the variant rs6502051 in *CCDC57*, which maps 3 Kb away from the top candidate *FASN* in WGHS (Eggert et al., 2012) was not reported in the Japanese GWAS. This SNP showed weak LD with our tested SNPs in *FASN* in EA and AA (Supplementary Tables S4, S5). The slightly different results obtained for this SNP (*P* = 0.13 in Table S1 and *P* = 0.029 in Table 3) stem from the use of models with (Table 1 and Table S1) and without (Table 3) adjustment for covariates.

Strong heterogeneity (*P* = 3.2 × 10^−4^) across the studies was also observed at the *THSD7B* variant rs4954368 in the test of association with the risk outcome (Table S3).

## Discussion

In the present NIEHS-UFS, we attempted replications of GWAS findings for uterine fibroids in the Japanese population and reported moderate and significant associations in race-stratified analyses and in meta-analyses depending on the SNP and outcome (risk or tumor size) under consideration. The most striking results were replicated associations of several variants in *TNRC6B* with either risk or tumor size. Based on the observed LD patterns across the associated SNPs, our data suggest the presence of two common *TNRC6B* haplotypes associated independently with risk or size of UL. Our data in NIEHS-UFS are consistent with the previous replication of *TNRC6B* SNP associations with risk and tumor size among European Americans in the BioVu and RFTS studies (Edwards et al., 2013a,b); however, differences between the association results from these replication studies were also observed. First, only few SNP associations could be compared because RFST/BioVU studies were focused on the three top GWAS genes (*BET1L, TNRC6B*, and near *SLK* genes) whereas the NIEHS-UFS examined all genome-wide significant SNPs. Among the rare overlapping SNPs in these replication studies *TNRC6B* rs12484776 was associated moderately with risk in RFTS and BioVu, and with increasing fibroid volume in RFTS whereas in NIEHS-UFS no association was observed with this SNP. The association observed in the meta-analysis of NIEHS-UFS and GWAS samples (Table 3) is solely explained by the latter samples. Second, while none of these studies reported evidence for *TNRC6B* rs139909 association with risk, association with tumor size (OR = 1.73; *p* = 0.008) was observed in the EA group in our study, potentially suggesting decreased power in the AA group that can be explained by the low MAF at this SNP.

We also reported moderate associations of intronic variant rs4954368 in *THSD7B* (thrombospondin, type I, domain containing 7B) with tumor size in race-stratified analyses and combined analyses of AA and EA in NIEHS-UFS but not in meta-analysis of risk in NIEHS-UFS and prior GWAS samples. The negative association with risk in the meta-analysis of the two studies is not inconsistent with the results from the separate studies, which showed no association in NIEHS-UFS and strong heterogeneity (*P* = 1.8 × 10^−5^) in the reported meta-analysis of the discovery and replication GWAS in the Japanese study (Cha et al., 2011). The reported association of *THSD7B* with tumor size may not be a spurious finding because (i) it was observed in a case-only design, (ii) the strength and direction of the association are similar in each study population, and (iii) the EA and AA groups share the allele “C” associated with tumor size.

A potential explanation to the observed association of rs4954368 with risk in the discovery GWAS but only with tumor size in NIEHS-UFS is the non-clinical ascertainment of the controls in the discovery GWAS. Non-clinical ascertainment of controls in cross-sectional studies of UL may be prone to high misclassification rates because women with small tumors are likely to be asymptomatic and consequently might not draw clinical attention. In the setting of high misclassification rates (large proportions of cases misclassified as controls), case and control designs would also test an ordinal outcome (small tumors vs. tumors with varying sizes). Thus, despite an appropriate and robust design of the reported GWAS (controls older than cases), misclassifications cannot be excluded. Alternatively, the negative association of *THSD7B* with risk and positive association with tumor size in NIEHS-UFS may reflect a pathogenic mechanism whereby the tagged causal variant in *THSD7B* is a secondary mutation rather than a driver mutation. Another possible explanation is the level of circulating estrogen, which affects the production of thrombospondins and may differ in NIEHS-UFS and the GWAS.

*THSD7B* encodes a member of the thrombospondins (TSP) which are potent inhibitors of angiogenesis and therefore may be important in controlling tumor growth (Vallbo and Damber, 2005). TSPs are secreted by various kinds of cells including endothelial and smooth muscle cells. These secreted molecules affect cell functions by modulating cell-matrix interactions. This finding converges with those from our recent study in NIEHS-UFS that implicated variants of encoded components of the extra-cellular matrix (Aissani et al., 2014).

We also reported that the intronic variant rs138039 in *TNRC6B* is associated with tumor size in EA with a significance level close to the Bonferroni-corrected threshold of *P* = 0.0009 and that the lower MAF in AA likely precluded detection of the association. The association of tumor size with a different *TNRC6B* variant (rs12484776) in RFTS (Edwards et al., 2013a), albeit moderately (*P* = 0.024), supports a candidate status for this gene because the observed association with a different variant in the same gene in RFTS reduces the possibility of biased results for rs138039 and rs139909 in NIEHS-UFS (not tested in RFTS). Given that these associated variants most likely tag rather than being the actual causal variant(s), cryptic difference in the pattern of linkage disequilibrium between the EA samples in NIEHS-UFS and RFTS or genetic heterogeneity (different causal variants) may explain the observed results.

We were not able to assess the replicated association with the *BET1L* 3′UTR variant rs2280543 that was reported in RFTS and BioVU; this variant did not pass the QC filter in our study. Nonetheless, the GWAS discovery variant rs11601352 in the adjacent *PSMD13* gene was shown to associate moderately with the risk of UL in AA and in meta-analysis in NIEHS-UFS (*P* = 0.042). The low frequency (MAF = 0.02) of the associated allele “A” at this SNP site, as well as that at rs2280543 in EA in RFTS and BioVU, precludes conclusive statements. Thus, validation of this association in other study populations with higher MAF is needed. Variant rs11601352 is not represented in the 1000 Genomes database and the highest MAF reported for this SNP in the HapMap III database is in populations of Mexican ancestry (MEX; MAF = 0.15), followed by Asian ancestries (China and Japan) with a MAF of 5%.

The function of *TNRC6B* is not known; however, this gene was highlighted as a candidate in GWAS for height (Estrada et al., 2009) and for age-at-menarche (Guo et al., 2006), which is a risk factor for UL (Marshall et al., 1997; Baird et al., 2003; Wise et al., 2004). Although no association was observed between *TNRC6B* variants and age-at-menarche in NIEHS-UFS (unpublished data), validation of the reported association in independent GWAS could imply that the encoded product of *TNRC6B* functions in the causal pathway. Of potential relevance to these independent replications of *TNRC6B* association with UL, the expression of the *TNRC6A* paralog was reported to be markedly dysregulated in large fibroids compared to small fibroids (log2 fold change = −1.00, *p* = 0.0008) (Guo et al., 2014). The *TNRC6* paralogs encode members of the GW182 protein family that are essential components of the miRNA pathway (Yao et al., 2012), which has been implicated in the pathogenesis of uterine fibroids (Marsh et al., 2008; Wei and Soteropoulos, 2008).

We also reported a moderate association of the common *CCDC57* variant rs6502051 with protection against UL in the AA group; however, the decreased level of significance for this SNP after controlling for the covariates suggested potential confounding effects. Furthermore, the negative association of the other common SNPs in this gene as well as in the nearby *FASN* with either outcome or study group remains unclear and requires confirmation in independent study populations. Finally, several SNP associations including those reported for *GPR19, HMGA2, MIAT, PSMD13/BET1L* and for other annotated or non-annotated gene loci had either low minor allele frequencies, were observed only in unadjusted models or that the association was lost or dropped significantly in meta-analysis of EA and AA or of EA, AA, and JPN samples.

The relevance of the encoded product of *THSD7B*, the sole gene that consistently associated with tumor size in EA and AA populations, to UL pathogenesis is not known. Very little is known about the function of this gene; it encodes a member of the thrombospondin family which are matricellular modulators of cell function (Bornstein, 2001). Of note, another member of the thrombospondin-encoding genes, *THBS1* (alias *TSP-1*), was reported to be down-regulated in uterine fibroids (Behera et al., 2007) and TSP-1 production has been shown to be directly controlled by estrogens in estrogen receptor-positive breast cancer cells (Hyder et al., 2009), suggesting that TSP-1 may function in the estrogen receptor-positive UL cells as well. Thus, the possibility that the product of *THSD7B* functions in the same pathway as TSP-1 cannot be excluded.

In conclusion, the present work replicated the association of *TNRC6B* with risk and size of UL consistent with the first reported replication of these associations in the RFTS and BioVU studies. Of potential relevance to the pathogenesis of UL, we reported an association of the thrombospondin-encoding *THSD7B* with tumor size in both European and African American populations.

## Author contributions

BA supervised the study design, data analyses and writing of the manuscript. KZ provided analytic support for the study design and data analyses. HW performed quality control of the data and statistical analyses. All authors read and approved the final version of the manuscript.

### Conflict of interest statement

The authors declare that the research was conducted in the absence of any commercial or financial relationships that could be construed as a potential conflict of interest.
