# Supplementary material for: Evaluation of GWAS candidate susceptibility loci for uterine leiomyoma in the multi-ethnic NIEHS uterine fibroid study
Source: Front Genet. 2015 Jul 14;6:241. doi: 10.3389/fgene.2015.00241 (PMC4501220; doi:10.3389/fgene.2015.00241)
Supplement: Supplementary file 4 [file Table4.DOCX]

**Supplementary Table S4. R-squared measures of linkage disequilibrium between intragenic SNPs in the European American group in NIEHS-UFS**

| ***HMGA2*** | rs2854603 | rs12423095 | rs2054547 | rs1563834 |
| --- | --- | --- | --- | --- |
| rs2854603 | 1 | 0.0007 | 0.3743 | 0.3749 |
| rs12423095 | 0.0007 | 1 | 0.1188 | 0.1189 |
| rs2054547 | 0.3743 | 0.1188 | 1 | 1 |
| rs1563834 | 0.3749 | 0.1189 | 1 | 1 |

| ***FASN-CCDC57*** | rs8066956 | rs12949488 | rs4246444 | rs6502051 | rs6502057 | rs11077969 | rs7406163 | rs7221544 | rs8080423 | rs7502078 | rs4247357 | rs4789698 |
| --- | --- | --- | --- | --- | --- | --- | --- | --- | --- | --- | --- | --- |
| rs8066956 | 1 | 0.0195 | 0.0765 | 0.0021 | 0.0018 | 0.0020 | 0.0023 | 0.0023 | 0.0023 | 0.0023 | 0.0025 | 0.0002 |
| rs12949488 | 0.0195 | 1 | 0.1037 | 0.1178 | 0.1279 | 0.1250 | 0.1221 | 0.1221 | 0.1221 | 0.1221 | 0.1306 | 0.2005 |
| rs4246444 | 0.0765 | 0.1037 | 1 | 0.2490 | 0.2094 | 0.2133 | 0.2172 | 0.2172 | 0.2172 | 0.2172 | 0.2062 | 0.1964 |
| rs6502051 | 0.0021 | 0.1178 | 0.2490 | 1 | 0.9312 | 0.9316 | 0.9316 | 0.9316 | 0.9316 | 0.9316 | 0.9180 | 0.8400 |
| rs6502057 | 0.0018 | 0.1279 | 0.2094 | 0.9312 | 1 | 1 | 1 | 1 | 1 | 1 | 0.9857 | 0.9021 |
| rs11077969 | 0.0020 | 0.1250 | 0.2133 | 0.9316 | 1 | 1 | 1 | 1 | 1 | 1 | 0.9858 | 0.9026 |
| rs7406163 | 0.0023 | 0.1221 | 0.2172 | 0.9316 | 1 | 1 | 1 | 1 | 1 | 1 | 0.9859 | 0.9031 |
| rs7221544 | 0.0023 | 0.1221 | 0.2172 | 0.9316 | 1 | 1 | 1 | 1 | 1 | 1 | 0.9859 | 0.9031 |
| rs8080423 | 0.0023 | 0.1221 | 0.2172 | 0.9316 | 1 | 1 | 1 | 1 | 1 | 1 | 0.9859 | 0.9031 |
| rs7502078 | 0.0023 | 0.1221 | 0.2172 | 0.9316 | 1 | 1 | 1 | 1 | 1 | 1 | 0.9859 | 0.9031 |
| rs4247357 | 0.0025 | 0.1306 | 0.2062 | 0.9180 | 0.9857 | 0.9858 | 0.9859 | 0.9859 | 0.9859 | 0.9859 | 1 | 0.8988 |
| rs4789698 | 0.0002 | 0.2005 | 0.1964 | 0.8400 | 0.9021 | 0.9026 | 0.9031 | 0.9031 | 0.9031 | 0.9031 | 0.8988 | 1 |

| ***TNRC6B*** | rs6001794 | rs11089974 | rs739182 | rs138039 | rs12484776 | rs139909 | rs2072858 | rs6001877 |
| --- | --- | --- | --- | --- | --- | --- | --- | --- |
| rs6001794 | 1 | 0.9466 | 0.4836 | 0.1637 | 0.7961 | 0.1234 | 0.4758 | 0.4752 |
| rs11089974 | 0.9466 | 1 | 0.5176 | 0.1784 | 0.8464 | 0.1345 | 0.5091 | 0.5087 |
| rs739182 | 0.4836 | 0.5176 | 1 | 0.3343 | 0.4870 | 0.2448 | 0.9502 | 0.9831 |
| rs138039 | 0.1637 | 0.1784 | 0.3343 | 1 | 0.1759 | 0.7363 | 0.3484 | 0.3362 |
| rs12484776 | 0.7961 | 0.8464 | 0.4870 | 0.1759 | 1 | 0.1293 | 0.5384 | 0.4881 |
| rs139909 | 0.1234 | 0.1345 | 0.2448 | 0.7363 | 0.1293 | 1 | 0.2588 | 0.2462 |
| rs2072858 | 0.4758 | 0.5091 | 0.9502 | 0.3484 | 0.5384 | 0.2588 | 1 | 0.9668 |
| rs6001877 | 0.4752 | 0.5087 | 0.9831 | 0.3362 | 0.4881 | 0.2462 | 0.9668 | 1 |
