# Supplementary material for: Evaluation of GWAS candidate susceptibility loci for uterine leiomyoma in the multi-ethnic NIEHS uterine fibroid study
Source: Front Genet. 2015 Jul 14;6:241. doi: 10.3389/fgene.2015.00241 (PMC4501220; doi:10.3389/fgene.2015.00241)
Supplement: Supplementary file 5 [file Table5.DOCX]

**Supplementary Table S5. R-squared measures of linkage disequilibrium between intragenic SNPs in the African American group in NIEHS-UFS**

| ***HMGA2*** | rs2854603 | rs12423095 | rs2054547 | rs1563834 |
| --- | --- | --- | --- | --- |
| rs2854603 | 1 | 0.0000 | 0.0006 | 0.0048 |
| rs12423095 | 0.0000 | 1 | 0.0265 | 0.0459 |
| rs2054547 | 0.0006 | 0.0265 | 1 | 0.6406 |
| rs1563834 | 0.0048 | 0.0459 | 0.6406 | 1 |

| ***FASN-CCDC57*** | rs8066956 | rs12949488 | rs4246444 | rs6502051 | rs6502057 | rs11077969 | rs7406163 | rs7221544 | rs8080423 | rs7502078 | rs4247357 | rs4789698 |
| --- | --- | --- | --- | --- | --- | --- | --- | --- | --- | --- | --- | --- |
| rs8066956 | 1 | 0.0022 | 0.0079 | 0.0037 | 0.0039 | 0.0025 | 0.0053 | 0.0049 | 0.0034 | 0.0009 | 0.0024 | 0.0261 |
| rs12949488 | 0.0022 | 1 | 0.1035 | 0.0112 | 0.0000 | 0.0003 | 0.0049 | 0.0055 | 0.0029 | 0.0021 | 0.0020 | 0.0076 |
| rs4246444 | 0.0079 | 0.1035 | 1 | 0.0361 | 0.0225 | 0.0182 | 0.0176 | 0.0148 | 0.0149 | 0.0067 | 0.0162 | 0.0270 |
| rs6502051 | 0.0037 | 0.0112 | 0.0361 | 1 | 0.6525 | 0.5985 | 0.5869 | 0.5499 | 0.5979 | 0.5325 | 0.5511 | 0.1816 |
| rs6502057 | 0.0039 | 0.0000 | 0.0225 | 0.6525 | 1 | 0.8940 | 0.8282 | 0.7748 | 0.8484 | 0.7896 | 0.8399 | 0.2205 |
| rs11077969 | 0.0025 | 0.0003 | 0.0182 | 0.5985 | 0.8940 | 1 | 0.9247 | 0.8551 | 0.9490 | 0.8799 | 0.7502 | 0.1515 |
| rs7406163 | 0.0053 | 0.0049 | 0.0176 | 0.5869 | 0.8282 | 0.9247 | 1 | 0.9850 | 0.9214 | 0.8240 | 0.6833 | 0.1300 |
| rs7221544 | 0.0049 | 0.0055 | 0.0148 | 0.5499 | 0.7748 | 0.8551 | 0.9850 | 1 | 0.8476 | 0.7624 | 0.6287 | 0.1213 |
| rs8080423 | 0.0034 | 0.0029 | 0.0149 | 0.5979 | 0.8484 | 0.9490 | 0.9214 | 0.8476 | 1 | 0.9038 | 0.7554 | 0.1575 |
| rs7502078 | 0.0009 | 0.0021 | 0.0067 | 0.5325 | 0.7896 | 0.8799 | 0.8240 | 0.7624 | 0.9038 | 1 | 0.6940 | 0.1311 |
| rs4247357 | 0.0024 | 0.0020 | 0.0162 | 0.5511 | 0.8399 | 0.7502 | 0.6833 | 0.6287 | 0.7554 | 0.6940 | 1 | 0.2675 |
| rs4789698 | 0.0261 | 0.0076 | 0.0270 | 0.1816 | 0.2205 | 0.1515 | 0.1300 | 0.1213 | 0.1575 | 0.1311 | 0.2675 | 1 |

| ***TNRC6B*** | rs6001794 | rs11089974 | rs739182 | rs138039 | rs12484776 | rs139909 | rs2072858 | rs6001877 |
| --- | --- | --- | --- | --- | --- | --- | --- | --- |
| rs6001794 | 1 | 0.7710 | 0.0146 | 0.0416 | 0.0365 | 0.0333 | 0.0036 | 0.0198 |
| rs11089974 | 0.7710 | 1 | 0.0177 | 0.0242 | 0.0505 | 0.0314 | 0.0067 | 0.0418 |
| rs739182 | 0.0146 | 0.0177 | 1 | 0.0090 | 0.1479 | 0.0052 | 0.5847 | 0.7360 |
| rs138039 | 0.0416 | 0.0242 | 0.0090 | 1 | 0.0244 | 0.7579 | 0.0159 | 0.0038 |
| rs12484776 | 0.0365 | 0.0505 | 0.1479 | 0.0244 | 1 | 0.0142 | 0.5410 | 0.1353 |
| rs139909 | 0.0333 | 0.0314 | 0.0052 | 0.7579 | 0.0142 | 1 | 0.0090 | 0.0108 |
| rs2072858 | 0.0036 | 0.0067 | 0.5847 | 0.0159 | 0.5410 | 0.0090 | 1 | 0.5624 |
| rs6001877 | 0.0198 | 0.0418 | 0.7360 | 0.0038 | 0.1353 | 0.0108 | 0.5624 | 1 |
