# Supplementary material for: Evaluation of GWAS candidate susceptibility loci for uterine leiomyoma in the multi-ethnic NIEHS uterine fibroid study
Source: Front Genet. 2015 Jul 14;6:241. doi: 10.3389/fgene.2015.00241 (PMC4501220; doi:10.3389/fgene.2015.00241)
Supplement: Supplementary file 6 [file DataSheet1.DOCX]

**Legends to supplementary Figure S1 and Tables S1-S5**

**Supplementary Figure S1: Principal Component Analysis showing clustering of the study populations of the National Institute of Environmental Health Science Uterine Fibroid Study (NIEHS-UFS) relative to the reference populations of the International HapMap III project**

The plot shows the results of Discriminant Analysis of Principal Components (DAPC) 1 and 2 to define clusters of genetically related individuals. A total set of 2,682 SNPs that were common to our data and to the majority of the HapMap III reference populations was used to assess the population membership of each of 1,003 individual of NIEHS-UFS (large blue diamonds) with genotyping call rates > 90%. The 3 ethnic groups (African Americans, European Americans and “other”) to which the sampled individuals self-identified clustered with the African (YRI, LWK and MKK) and African American (ASW) populations, the European populations and the more scattered group composed of Asians, Hispanics and individuals of mixed origin, respectively.

**Supplementary Table S1: Association of SNPs with the risk of uterine fibroids in the NIEHS uterine fibroid study**

(a) List of candidate single nucleotide polymorphisms (SNP) from genome-wide association studies (GWAS) of uterine leiomyoma (UL) in populations of Japanese ([Cha et al. 2011](#_ENREF_12)) or European ([Eggert et al. 2012](#_ENREF_18)) ancestries. SNPs showing significant associations with risk of UL at the 5% cut-off in NIEHS-UFS (National Institute of Environmental Health Sciences uterine fibroid study) are highlighted in bold. (b) alternate (A1) and reference (A2) alleles. (c) alternate allele frequency in African Americans (AA) and European Americans (EA). (d) Chromosome. Blanks in the column with the heading “discovery GWAS” indicate SNPs associated with fibroids in previous candidate gene approach studies. (e) In few instances where the SNPs locate in extended intergenic regions, the closest genes are shown.(f) Logistic regression analysis assuming an additive genetic model. (g) Odds ratios (OR) and 95% confidence intervals (95% CI) from logistic regression models adjusted for age, age at menarche, parity after age 25, body mass index and physical activity. Meta-analysis was conducted in the metaphor R package using random-effect models ([Viechtbauer 2010](#_ENREF_36)). P value for heterogeneity (*P*-het) across the samples.

**Supplementary Table S2: Association of SNPs with the size of uterine fibroids in the NIEHS uterine fibroid study**

(a) List of candidate single nucleotide polymorphisms (SNP) from genome-wide association study (GWAS) of uterine leiomyoma (UL) in populations of Japanese ([Cha et al. 2011](#_ENREF_12)) and European ([Eggert et al. 2012](#_ENREF_18)) ancestries. SNPs showing significant associations with tumor size at the 5% cut-off in NIEHS-UFS (National Institute of Environmental Health Sciences uterine fibroid study) are highlighted in bold. (b) alternate (A1) and reference (A2) alleles . (c) alternate allele frequency in European Americans (EA) and African Americans (AA). (d) Chromosome. Blanks in the column with the heading “discovery GWAS” indicate SNPs associated with risk of UL in previous candidate gene approach studies. (e) In few instances where the SNPs locate in extended intergenic regions, the closest genes are shown. (f) logistic regression analysis assuming an additive genetic model. (g) Odds ratios (OR) and 95% confidence intervals (95% CI) from proportional odds models adjusted for age, age-at-menarche, parity, BMI and physical activity. Meta-analysis was conducted in the metaphor R package using random-effect models ([Viechtbauer 2010](#_ENREF_36)). Because neither discovery GWAS studied the tumor size outcome, only the NIEHS AA and EA samples were included in the meta-analysis. Note that the underlined SNP rs1481045 did not meet the assumption of proportional odd. Blanks refer to SNP models that did not converge.

**Supplementary Table S3: Meta-analysis of risk for UL in African American, European American and Japanese populations (n=10,662)**

(a) List of candidate single nucleotide polymorphisms (SNP) from genome-wide association study (GWAS) data for uterine leiomyoma (UL) in populations of Japanese ancestry ([Cha et al. 2011](#_ENREF_12)) or European American ancestry enrolled in the Women’s Genome Health Study (WGHS) ([Eggert et al. 2012](#_ENREF_18)), and from UL candidate gene *HMGA2* (high mobility group AT-hook 2). SNPs that showed significant associations at the 5% cut-off with risk in race-stratified logistic regression models in African Americans (AA) or European Americans (EA) in NIEHS-UFS (National Institute of Environmental Health Sciences uterine fibroid study) and/or in meta-analysis of AA and EA are highlighted in bold. (b) Chromosome. (c) alternate (A1) and reference (A2) allele. (d) alternate allele frequency in AA, EA and Japanese (JPN). The beta coefficients (beta) and standard errors (SE) used in the meta-analysis were derived from the present study for NIEHS-UFS and by conversion of the estimated odds ratios and 95% confidence intervals in the discovery GWAS and replication study in the Japanese study. Estimates of beta and SE from WGHS were not available for the present study; therefore for the SNPs studied only NIEHS-UFS and WGHS, the results of meta-analysis are those reported in Table 1 for NIEHS-UFS. Meta-analysis was conducted in the metaphor R package ([Viechtbauer 2010](#_ENREF_36)) using random-effect models and Dersimonian-Laird estimator. (e) p-value for heterogeneity (*P*-het) across samples.

**Supplementary Table S4. R-squared measures of linkage disequilibrium between intragenic SNPs in the European American group in NIEHS-UFS.**

The Table shows measures of the strength of linkage disequilibrium (correlation coefficient r^2^ ) between single nucleotides polymorphisms (SNPs) located in genes associated with risk or diameter size of Uterine Leiomyoma (UL) in the study group of European American descent. *HMGA2* (high mobility group AT-hook 2); FASN (fatty acid synthase); *CCDC57* (coiled-coil domain containing 57); *TNRC6B* (trinucleotide repeat containing 6B).

**Supplementary Table S5. R-squared measures of linkage disequilibrium between intragenic SNPs in the African American group in NIEHS-UFS**

The Table shows measures of the strength of linkage disequilibrium (correlation coefficient r^2^ ) between single nucleotides polymorphisms (SNPs) located in genes associated with risk or diameter size of Uterine Leiomyoma (UL) in the study group of African American descent. *HMGA2* (high mobility group AT-hook 2); FASN (fatty acid synthase); *CCDC57* (coiled-coil domain containing 57); *TNRC6B* (trinucleotide repeat containing 6B).
