# Supplementary figures and images for: Evaluation of GWAS candidate susceptibility loci for uterine leiomyoma in the multi-ethnic NIEHS uterine fibroid study
Source: Front Genet. 2015 Jul 14;6:241. doi: 10.3389/fgene.2015.00241 (PMC4501220; doi:10.3389/fgene.2015.00241)

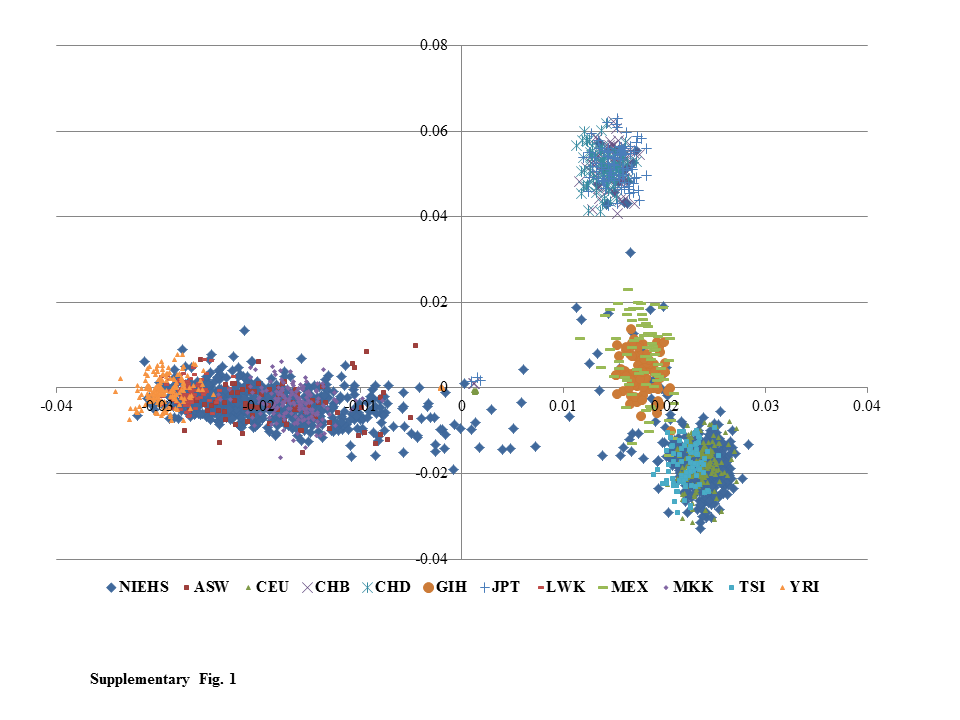

Supplement: Supplementary file 7 [file Image1.TIF]
